# Benzene poisoning, clinical and blood abnormalities in two Brazilian female gas station attendants: two case reports

**DOI:** 10.1186/s13104-016-2369-8

**Published:** 2017-01-18

**Authors:** Fábio Santiago, Simone Lima, Tayná Pinheiro, Rafaele Tavares Silvestre, Ubirani Barros Otero, Marianne Medeiros Tabalipa, Nadezda Kosyakova, Maria Helena Ornellas, Thomas Liehr, Gilda Alves

**Affiliations:** 1 Laboratório de Marcadores Circulantes, Departamento de Patologia e Laboratórios, Faculdade de Ciências Médicas, Universidade do Estado do Rio de Janeiro, Rio de Janeiro, Brazil; 2 Pós-graduação em Ciências Médicas (PGCM), Universidade do Estado do Rio de Janeiro, Rio de Janeiro, Brazil; 3 Coordenação de Pesquisa, Instituto Nacional de Câncer, Rio de Janeiro, Brazil; 4 Unidade Técnica de Exposição Ocupacional, Ambiental e Câncer, Coordenação de Prevenção e Vigilância, Instituto Nacional de Câncer, Rio de Janeiro, Brazil; 5 Jena University Hospital, Friedrich Schiller University, Institute of Human Genetics, Kollegiengasse 10, D-07743 Jena, Germany; 6 Departamento de Patologia Geral, Faculdade de Ciências Médicas, Universidade do Estado do Rio de Janeiro, Avenida Professor Manuel de Abreu 444, 4° andar, Vila Isabel, Rio de Janeiro, 20551-030 Brazil

**Keywords:** Benzene, Toluene, Xylene, Cytogenetic, Painting chromosome, Natural killer

## Abstract

**Background:**

Brazilian gas station workers are chronically exposed to benzene, toluene, xylene (BTX) during their working time. Describe below two cases of latin female gas station workers with benzene poisoning symptoms and miscarriage history.

**Case presentation:**

In both cases were identified complex chromosomal rearrangements (CCR) with fluorescence in situ hybridization, applied to whole chromosome paints by chromosomes 1, 2 and 4. The lower natural killer cell (NK) cells have also been observed in cases correspondents, especially the rare type of NK (NKbright) in their peripheral blood cells.

**Conclusions:**

It is known that acquired chromosomal aberrations are positively correlated with cancer and reproductive risk. In concordance, lower NK cytotoxicity increases the risk for cancer, as well. Thus, this is the first study providing hints on a possible causative relation of lower NK cytotoxicity and increase rates of chromosomal rearrangements including CCRs.

## Background

Brazilian gas station workers are chronic exposed to benzene, toluene and xylene (BTX), mainly benzene, during the working time [1]. Chronic exposure to benzene may lead to progressive degeneration of bone marrow, aplastic anemia and/or leukemia. According to the U.S. Department of Labor only a detailed history and appropriate investigative procedures will enable a physician to rule out or confirm the benzene poisoning [2]. To assist the examining physician the cytogenetic tests with fluorescence in situ hybridization (FISH) shown to be a new and valuable tool to determine the workers in risk [3]. Genetic damages caused by benzene include sister chromatid exchanges, DNA cross linking agents, DNA adduct formations, and impairment of DNA repair mechanisms [4].

Since 2010, 115 gas station attendants have been monitored in Rio de Janeiro city, Brazil applying FISH, using whole chromosome painting (wcp) probes for chromosomes 1, 2 and 4. Among the study group, the medical inquiry identified two female gas station attendants with signs and symptoms of acute benzene intoxication, associated with history of abortions. Added to the cytogenetic tests an large hematological evaluation by cytometry were made and found a down regulation of the natural killer (NK) cells association an acquired complex chromosomal rearrangements (CCRs).

## Case presentation

### Case 1

**Fig. 1:**
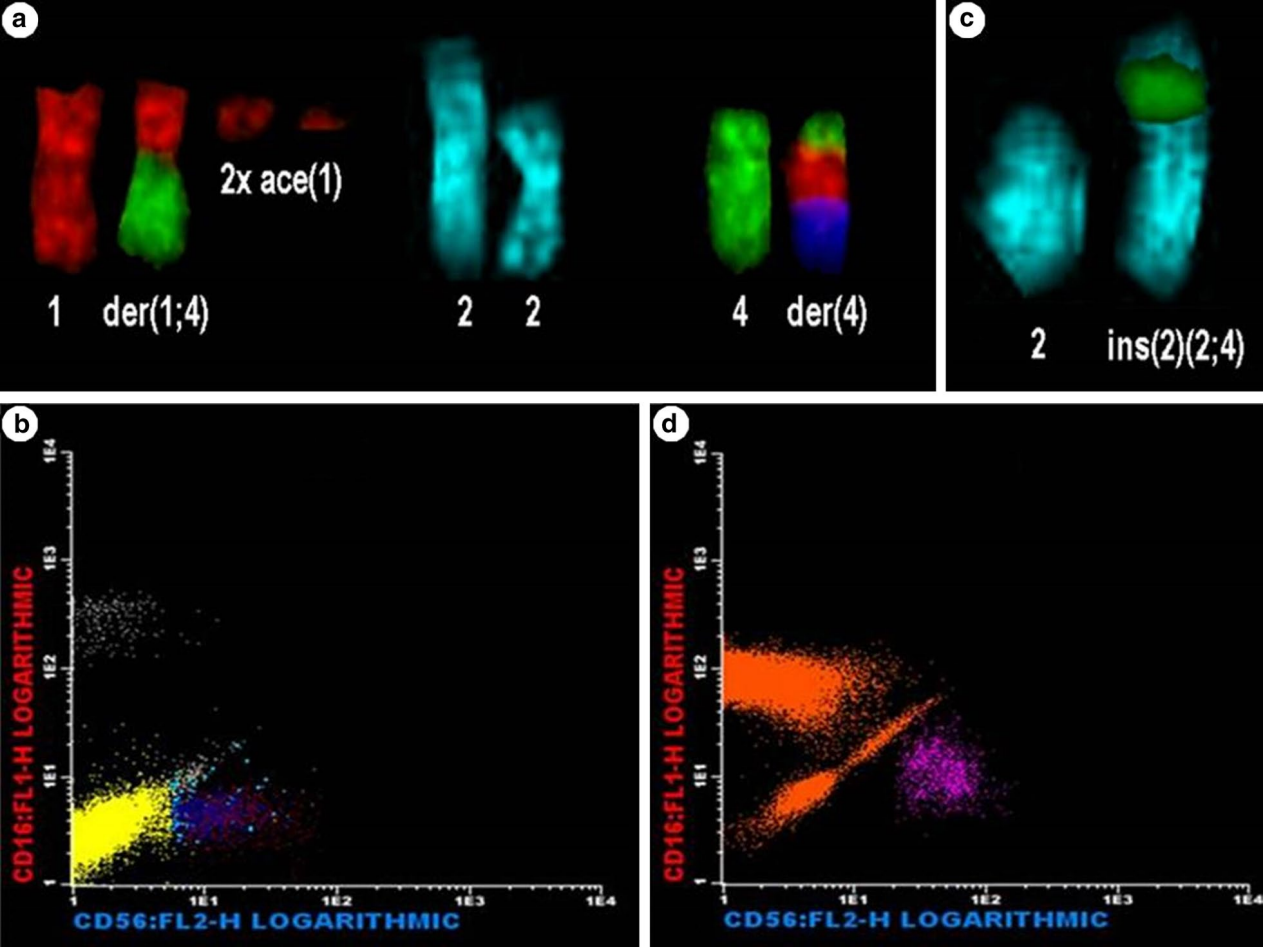
CCRs found in case 1 and 2 and flow cytometric analyses of NK cell subsets. Case 1: **a** CCR—der(1)t(1;4),der(4)t(1;4;?),ace(1),ace(1). **b**, flow cytometric analyses of NK cell of case 1. Show all NK cells (CD56 positive) are CD16 negative, thus all are sub-sets of NK^bright^. Case 2: **c** CCR- ins(2;4). Chromosome 2 was folded and thus looks shorter due to preparation. **d** flow cytometric analyses of NK cell of case 1. Show all NK cells (CD56 positive) are CD16 negative, thus all are sub-sets of NK^bright^. **a**, **b** the probes were conjugated with TexasRed to label chromosome 1 (*red*), Diethylaminocoumarin (DEAC) for chromosome 2 (*lightblue*) and fluorescein isothiocyanate (FITC) for chromosome 4 (*green*). Other chromosomes were counterstained with DAPI (*dark blue*). **c**, **d** BD FACSCanto II cytometer was used in both cases and the data analyses were performed using the Infinicyt^®^ software

A 25-year-old latin woman, working 8 h per day, 6 days a week, for the last 4 years as a gas station attendant had one gestation with miscarriage in the first half of pregnancy. Headache, dizziness irritability, asthenia and normal menstrual cycle were reported. A physical examination showed changes in the thyroid gland, nodules in the right lobe, and a nonspecific pulmonary auscultation. The attendant also reported being a former smoker and not having a family history of cancer. Was found in 1/100 metaphases a CCR involving 8 chromosomal breakpoints described as: 46,XX,der(1)t(1;4),der(4)t(1;4;?),ace(1),ace(1), (Fig. 1). Hemogram showed mild neutopenia (1470 cells/mm^3^) and biochemistry tests revealed no changes compared to normal values as described in Table 1. On the other hand, the immunophenotypic analysis confirmed neutropenia (33.00%), with a lower NK cell count (2.28%), with all NK CD56+/CD16− (Table 2; Fig. 1).

**Table 1:** Biometrics data (clinical and demographic) of 2 female gas station attendants

Biometrics data	Case 1	Case 2
Smokers	No	No
Ex-smokers	Yes	No
Illicit drug consumption	Yes^a^	No
Alcohol abuse	Yes	No
Family history of cancer	No	No
Blood test		
Erythrocytes (10^6^/µL)	4.0	4.2
Hemoglobin (g/dL)	12.0	12.5
Hematocrit (%)	36.1	38.0
Mean corpuscular volume (fl)	88.7	88.6
Leukocytes (/uL)	4620	7410
Neutrophils (%)	32.1	57.4
Typical lymphocytes (%)	56.7	34.5
Basophils (%)	0.6	0.4
Eosinophils (%)	3.7	1.5
Monocytes (%)	6.9	6.2
Platelets (10^3^/µL)	230	312
Reticulocytes (%)	1.21	0.7
Gamma-GT (U/L)	31.0	14.0
Oxaloacetic transaminase (U/L)	0.23	17.0
Pyruvic transaminase (U/L)	15.0	11.0
Creatinine (mg/dL)	0.6	0.7
Total bilirubin (mg/dL)	0.23	0.38
Lactate dehydrogenase (mg/dL)	252	320
Rheumatoid factor (IU/mL)	9.6	9.5
C reactive protein (mg/dL)	0.11	0.12

*Gamma GT* Gamma glutamyl transpeptidase

^a^Marijuana

**Table 2:** Results of Immunophenotyping by flow cytometry

Monoclonal antibody	Lymphocyte	Monocyte	Granulocyte	Eosinophil
Case 1				
Percent of total cells	54.1	6.7	33.00	3.03
CD 4	28.56	–	–	–
CD 8	13.75	–	–	–
CD4 + CD 8	42.31	–	–	–
CD5	44.20	–	–	–
CD7	50.00	–	–	–
CD2	46.60	–	–	–
CD3	41.87	–	–	–
CD16	0.00	0.00	36.5	0.00
CD56	2.28	0.00	0.00	0.00
CD16 + CD 56 (NK)	2.28	0.00	0.00	0.00
CD19	5.77	–	–	–
CD20	6.10	–	–	–
CD10	0.07	–	28.94	–
CD27	0.00	0.00	0.00	3.52
CD22	5.88	0.00	0.00	0.00
HLA-DR	7.23	6.42	0.00	4.00
CD11c	3.08	7.06	32.4	3.52
CD25	0.00	0.00	0.00	5.70
Case 2				
Percent of total cells	31.23	5.4	63.6	0.16
CD 4	13.51	–	–	–
CD 8	9.08	–	–	–
CD4 + CD8	22.59	–	–	–
CD5	30.85	–	–	–
CD7	25.17	–	–	–
CD2	24.36	–	–	–
CD3	22.59	–	–	–
CD16	0.00	0.00	55.06	0.00
CD56	1.51	0.00	0.00	0.00
CD16 + CD 56 (NK)	1.51	0.00	0.00	0.00
CD19	5.58	–	–	–
CD20	5.59	–	–	–
CD10	0.13	–	46.09	–
CD27	0.00	0.00	0.00	0.00
CD22	4.88	0.00	0.00	0.00
HLA-DR	4.89	7.12	0.00	0.00
CD 11c	0.20	3.75	49.12	0.00
CD25	0.00	0.00	0.00	0.00

### Case 2

A 40-year-old latin woman, who was not a smoker or drug addict, working 48 h a week for the last 9 years as a gas station attendant had a pathological history of one miscarriage in the first half of pregnancy. Anxiety, dizziness, cramps, asthenia and normal menstrual cycle were reported. No physical examination alteration and no family history of cancer were observed. For case 2, Fig. 1 shows one CCR, which was found in 1/100 metaphases, described as der(4)ins(2;4), which was due to a 3 breakpoint event. Complete hemogram and biochemistry tests showed no abnormalities in comparison with normal values, as described in Table I. Like case 1, the immunophenotypic analysis of case 2 resulted in a lower NK cell count (1.51%), with all NK CD56+/CD16− (Table 2; Fig. 1).

## Conclusions

We characterized cytogenetic, hematological, and immunophenotypic status in two female gas station attendants, who working in gas station with a proved harmful enviromental concentration of BTX. The following abnormalities were found: CCRs, a decrease in NK cells with abnormal CD16 expression, and early pregnancy loss.

It is well known that gas station workers are exposed to potentially harmful chemicals including BTX. However, benzene is considered the main carcinogenic agent (group 1 according to IARC) and studies associate this compound with acquired cytogenetic alterations [5–7]. Among various forms of benzene-induced genetic alterations, aneuploidy and chromosomal breakage are the most studied [4]. Chromosomal aberrations in peripheral blood lymphocytes of chronically benzene-exposed patients were previously documented [5–7]. Zhang et al. [5] reported dose-dependent chromosomal aneuploidies (mono- and trisomies) in the peripheral blood lymphocytes of workers exposed to benzene. In our study, we analyzed CAs only in three pairs of chromosomes, which make up 22.8% of the human genome. It’s a cheaper and faster test to estimate the DNA damage when compared to whole genome CA screening. Chromosomal aberrations (CAs) of high complexities could be detected in 1 out of 100 metaphases per patient (i.e. 1%). The rate of CCRs in normal controls lies between 0 and 0.5%, determined in 1000 metaphases, each [8]. In the present study, only 100 metaphases could be analyzed per case. Thus, the finding of one meta- phase with a CCR among 100 cells is at least noteworthy.

Even though CCR detection in peripheral blood is not directly correlated with enhanced cancer risk, it should be kept in mind that such CAs may indicate increased radio- and/or chemosensitivity. As tumors may be induced by environmental factors in combination with a special genetic susceptibility, the two cases reported may be at risk of acquiring malignancies [8].

As is well known, meiosis is a complex process controlled by different checkpoints, but males and females respond differently to meiotic disturbances [9]. During oogenesis, meiosis is generally pursued leading to the formation of aneuploid gametes or with single gene mutations.

Thus, in gametes, acquired genetic changes can be passed on to the next generation. Several epidemiological studies support the idea that genotoxic and nongenotoxic events following benzene exposure may be initiators of childhood leukemia in utero [10]. Another study on AML has shown that disease is usually initiated in utero because the leukemic translocations and other genetic changes are present in blood spots collected at birth [10, 11]. Also interesting is the fact that the majority of the CCR cases are reported in females ascertained through repeated spontaneous abortions or the birth of a malformed child [11].

Besides the detected CCRs pointing towards enhanced chemosensitivity, these two female workers had hematological and immunological abnormalities characterized by mild leukopenia (case 1) and NK abnormalities. There are some studies concerning benzene with hematological and immunological abnormalities in humans [12–16]. The effects of immunotoxicity induced by benzene are depression and alteration of both the immune system mediated by cells and the humoral system [15]. Lan et al. [13] observed that leucocytes, B and CD4+-T cell counts, were significantly decreased in workers exposed to benzene compared to the controls. In another study, the number of T lymphocytes, lymphocytes T CD4 and T CD8, and NK cells was reduced in the percentages and absolute numbers, and an increase in the monocyte count in workers during the period of exposure was found [15]. Thus, it was suggested that the depressive effect of benzene on the T and NK cells may be a factor of the probable carcinogenic activity of benzene through the immune system.

Natural killer (NK) cells are immune effector cells that recognize both virally infected and malignant target cells. Surprisingly, the results of the immunophenotypic analysis revealed NK CD56 positive (normal fluorescence) and CD16 negative in both cases, suggesting the presence of the rare subtype NK bright in the peripheral blood, which has low cytotoxic action [15]. It is possible that the action of BTX on the immune system had blocked the transition of immature CD56 bright cells into CD56 dim cells. In agreement with this finding, an 11 year follow up study showed that low NK cytotoxicity of peripheral blood lymphocytes correlates with an increased risk for cancer [16].

The identifications of chromosomal abnormalities and NK downregulation in the blood may be a new indicator for effective follow up of workers exposed to BTX, preventing diseases mainly important for females and their offspring. Further studies with a larger number of workers are necessary to confirm the results found.

## Abbreviations

AML: acute myeloid leukemia
BTX: benzene, toluene and xylene
CAs: chromosomal aberrations
CCR: complex chromosomal rearrangements
FISH: fluorescence in situ hybridization
NK: natural killer
